# Supplementary material for: Nuclear–Cytoplasmic Shuttling of the Usher Syndrome 1G Protein SANS Differs from Its Paralog ANKS4B
Source: Cells. 2024 Nov 8;13(22):1855. doi: 10.3390/cells13221855 (PMC11592671; doi:10.3390/cells13221855)
Supplement: Supplementary file 1 [file cells-13-01855-s001.zip › Supplemenatry_Files/Supplementary_Figure_File_revised.pdf]

Supplementary Material

Nucleocytoplasmic shuttling of the Usher syndrome 1G protein SANS differs from its paralogue ANKS4B

Jacques S. Fritze[1], Felizitas F. Stiehler[1] and Uwe Wolfrum[1#]

[1]Institute of Molecular Physiology, Johannes Gutenberg University Mainz, Mainz, Germany

[#] Corresponding author: Uwe Wolfrum
wolfrum@uni-mainz.de

1. Supplementary Figures and Tables

Supplementary_Figure_File.docx: all Figures S1-S9 referred to in this work;

Table_S1.xlsx: GO-term analysis of SANS nuclear interactome from (Yildirim et al. 2021)
Table_S2.xlsx: Missense3D prediction of $SANS^{K213E}$ and $SANS^{L195E}$
Table_S3: Predicted nuclear export sequences (NES) of ANKS4B

1.1 Supplementary Figures

Figure S1:

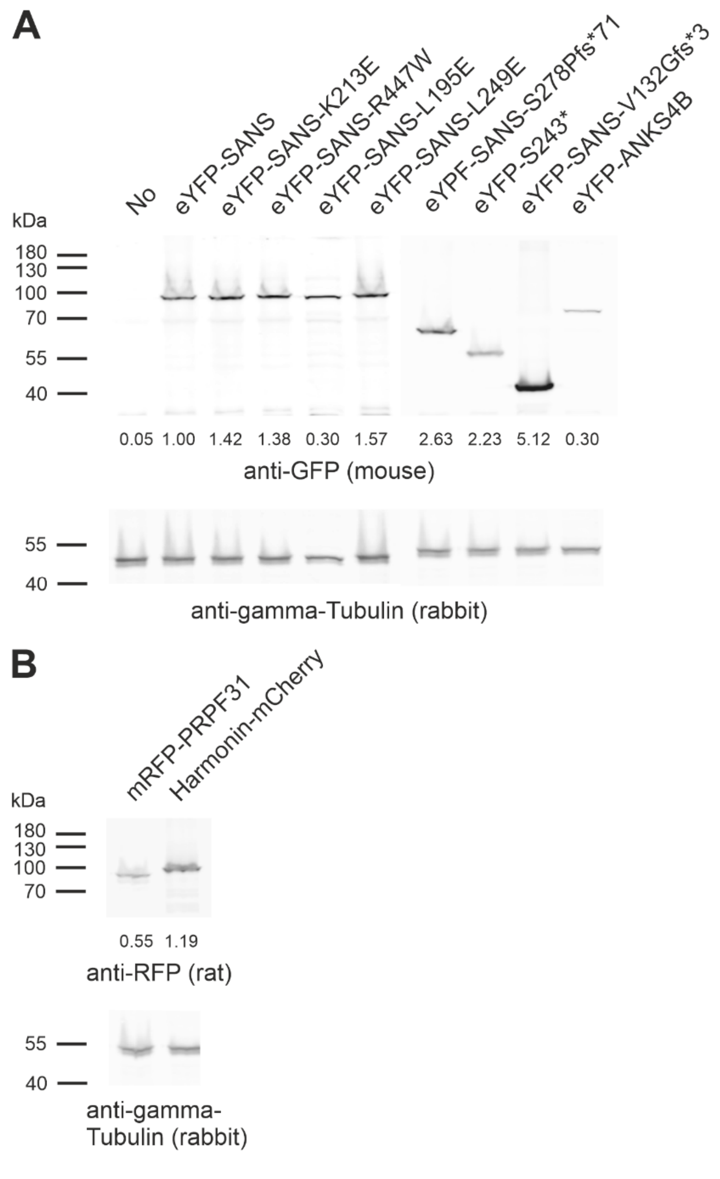

Figure S1. Western blot analysis of tagged constructs used. (A, B) Anti-eYFP **(A)** or anti-RFP **(B)** Western blot analysis of lysates of HeLa cells expressing different eYFP-SANS, eYFP-ANKS4B, mRFP-PRPF31, or harmonin-mCherry constructs. All transfected constructs are detected with the expected size (eYFP-SANS and eYFP-SANS-K213E, -R4447W, -L195E, -L249E ~80 kDa; eYFP-SANS^{S278Pfs*71} ~66 kDa; eYFP-SANS^{S243*} ~56 kDa; eYFP-SANS^{V132Gfs*3} ~42 kDa; eYFP-ANKS4B ~75 kDa; mRFP-PRPF31 ~82 kDa; harmonin-mCherry ~88 kDa). All constructs were detectable by the corresponding antibody. For the initial SDS-page 30 µg of protein was loaded. Band intensity (numbers) is normalized to Tubulin and set to 1 for eYFP-SANS.

Figure S2:

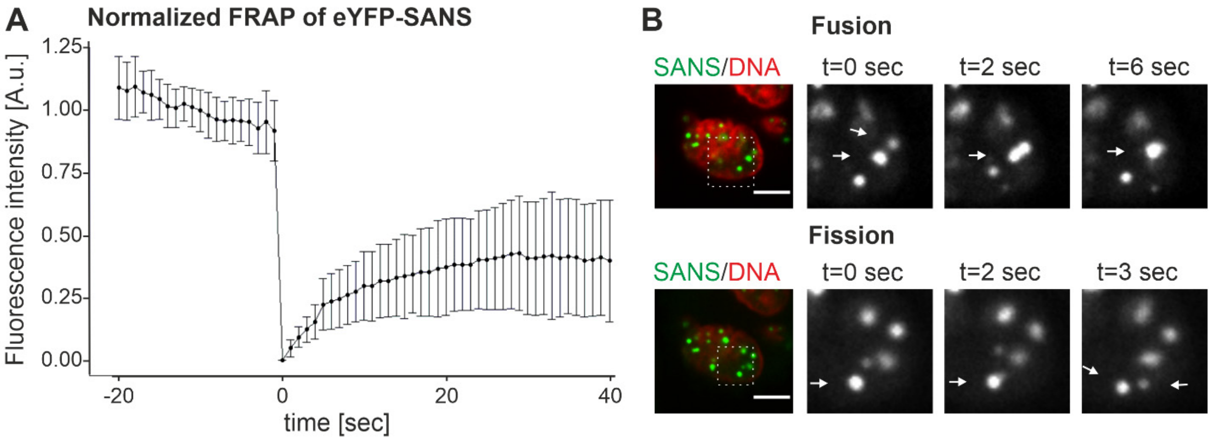

Figure S2. Analysis of eYFP-SANS speckle properties by fluorescence recovery after photobleaching (FRAP) and live cell imaging. (A) eYFP-SANS speckles were bleached with 36% laser intensity for fluorescence recovery after photobleaching (FRAP). eYFP-SANS recovered with a half-time of 39.9 sec. to ~46% of its original intensity. **(B)** eYFP-SANS speckles separated and fussed in a timeframe of seconds, which indicates a high mobility of these speckles.

Figure S3

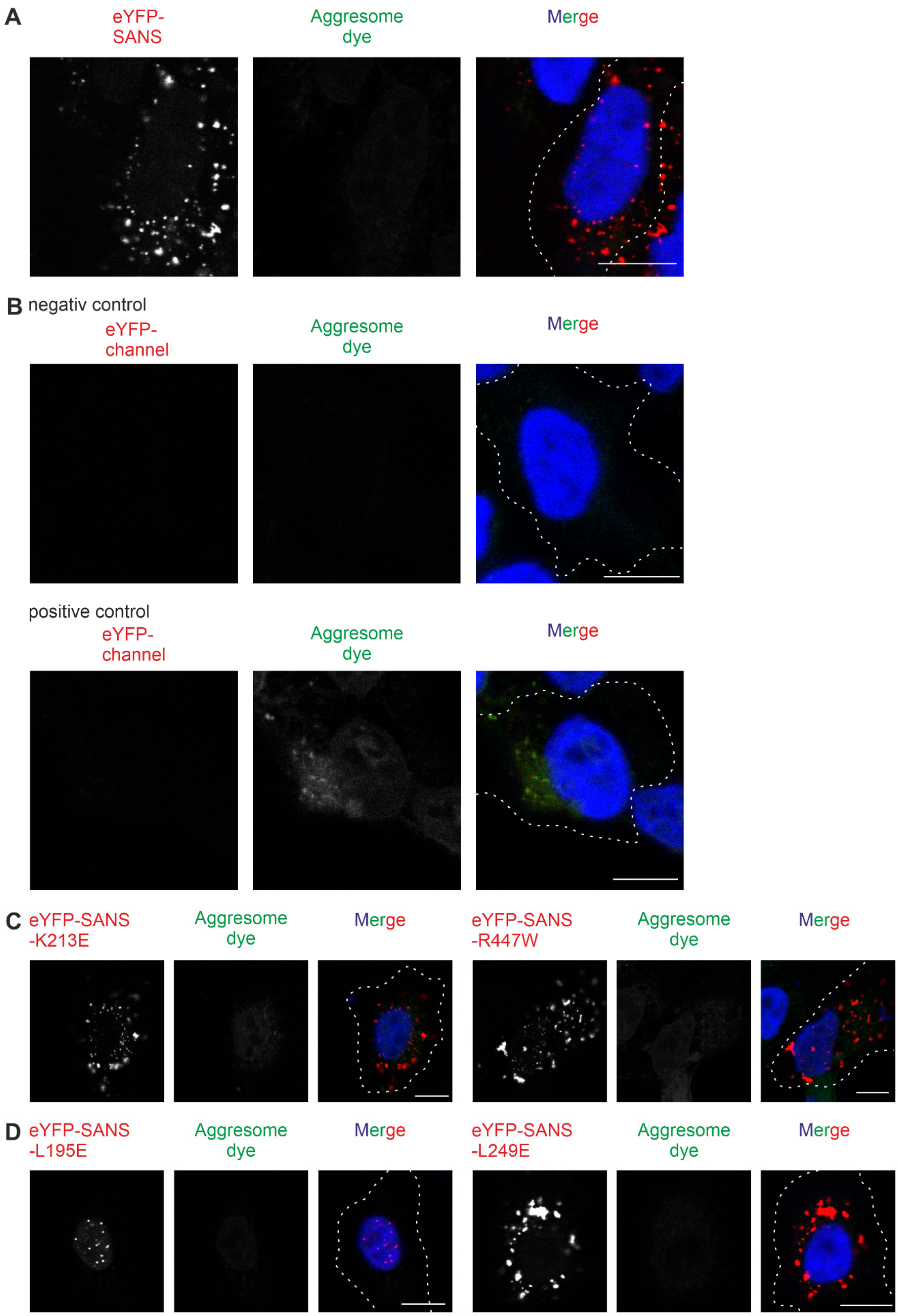
A
eYFP-SANS
Aggresome dye
Merge
B negativ control
eYFP-channel
Aggresome dye
Merge
positive control
eYFP-channel
Aggresome dye
Merge
C eYFP-SANS -K213E
Aggresome dye
Merge
eYFP-SANS -R447W
Aggresome dye
Merge
D eYFP-SANS -L195E
Aggresome dye
Merge
eYFP-SANS -L249E
Aggresome dye
Merge

Figure S3. Fluorescence microscopic analysis of eYFP-SANS speckles by cytochemical staining for aggresomes. (A-D) HeLa cells were treated with DMSO (negative control), MG-132 (positive control) or transfected with eYFP-SANS and its mutants. Aggresome staining was performed with a commercial kit and cells were counterstained with DAPI. Transfected cells do not show an aggresome staining. Scale bar = 10 µm. Pearson coefficient R values do not indicate co-localization.

Figure S4:

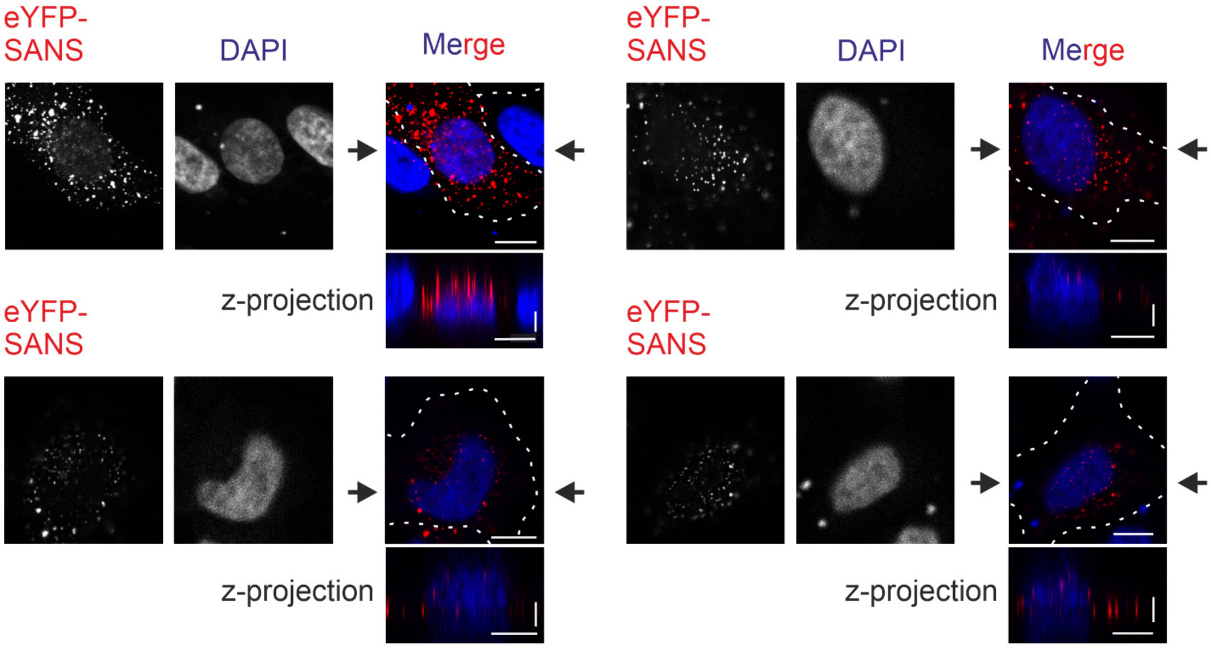

Figure S4. Subcellular localization of eYFP-SANS in HeLa cells. Additional confocal microscopy of HeLa cells transfected with eYFP-SANS (red) counterstained with DAPI related to Figures 1. Black arrows: position of Z-projections. Scale bars: horizontal = 10 µm; vertical = 2 µm.

Figure S5:

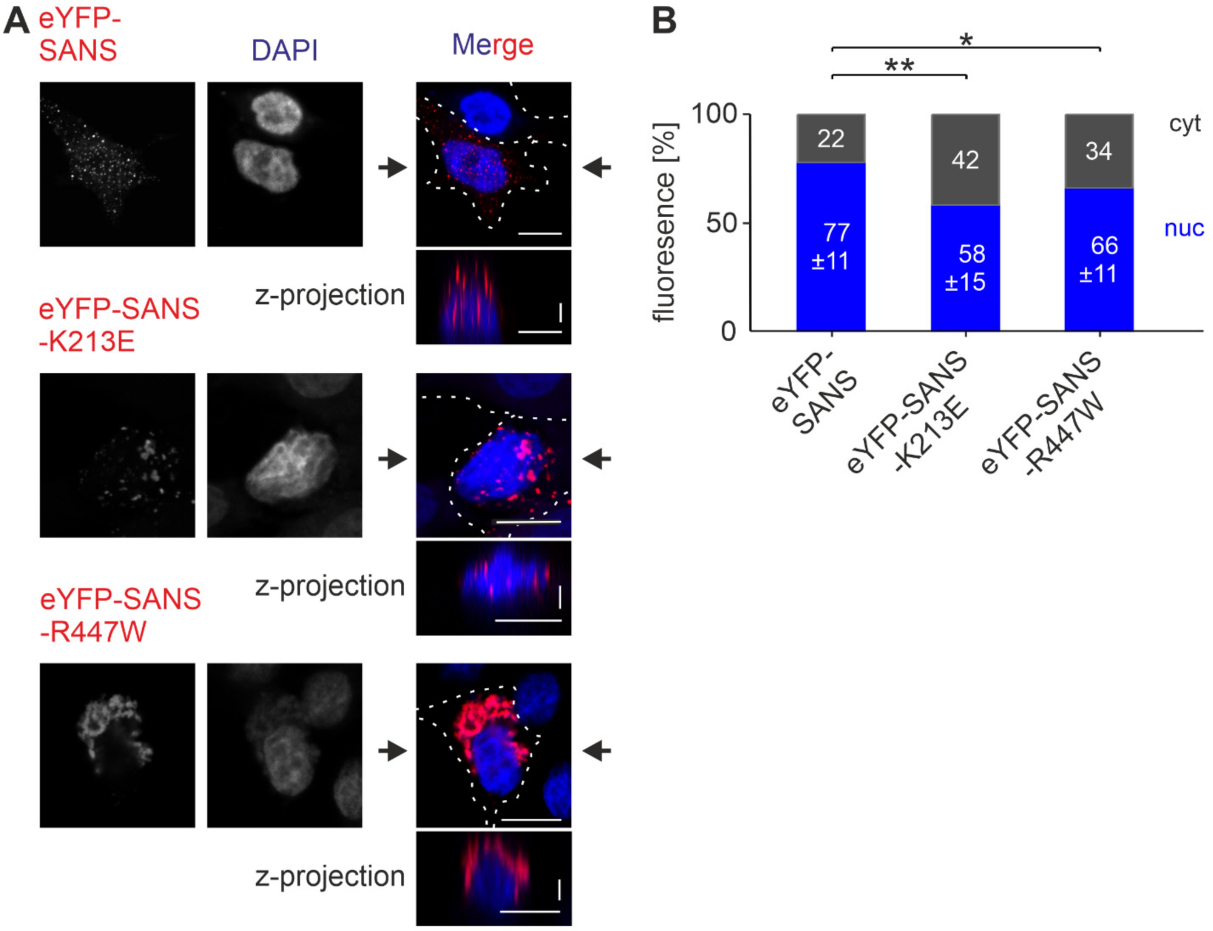

Figure S5. Subcellular localization of SANS and NLS mutants in HEK293T cells. (B) Confocal microscopy of HEK293T cells transfected with eYFP-SANS (red) or SANS NLS mutants, counterstained with DAPI. **(B)** Quantification of (A) by CellProfiler. eYFP-SANS^{K213E} and eYFP-SANS^{R447W} differed significantly from eYFP-SANS. Black arrows: position of Z-projections. Scale bars: horizontal = 10 µm; vertical = 2 µm. Data show mean values ± standard deviation from three independent experiments. Students t-test was performed for 3 independent experiments with a minimum of 75 cells; * = p < 0.05; . * = p < 0.009.

Figure S6:

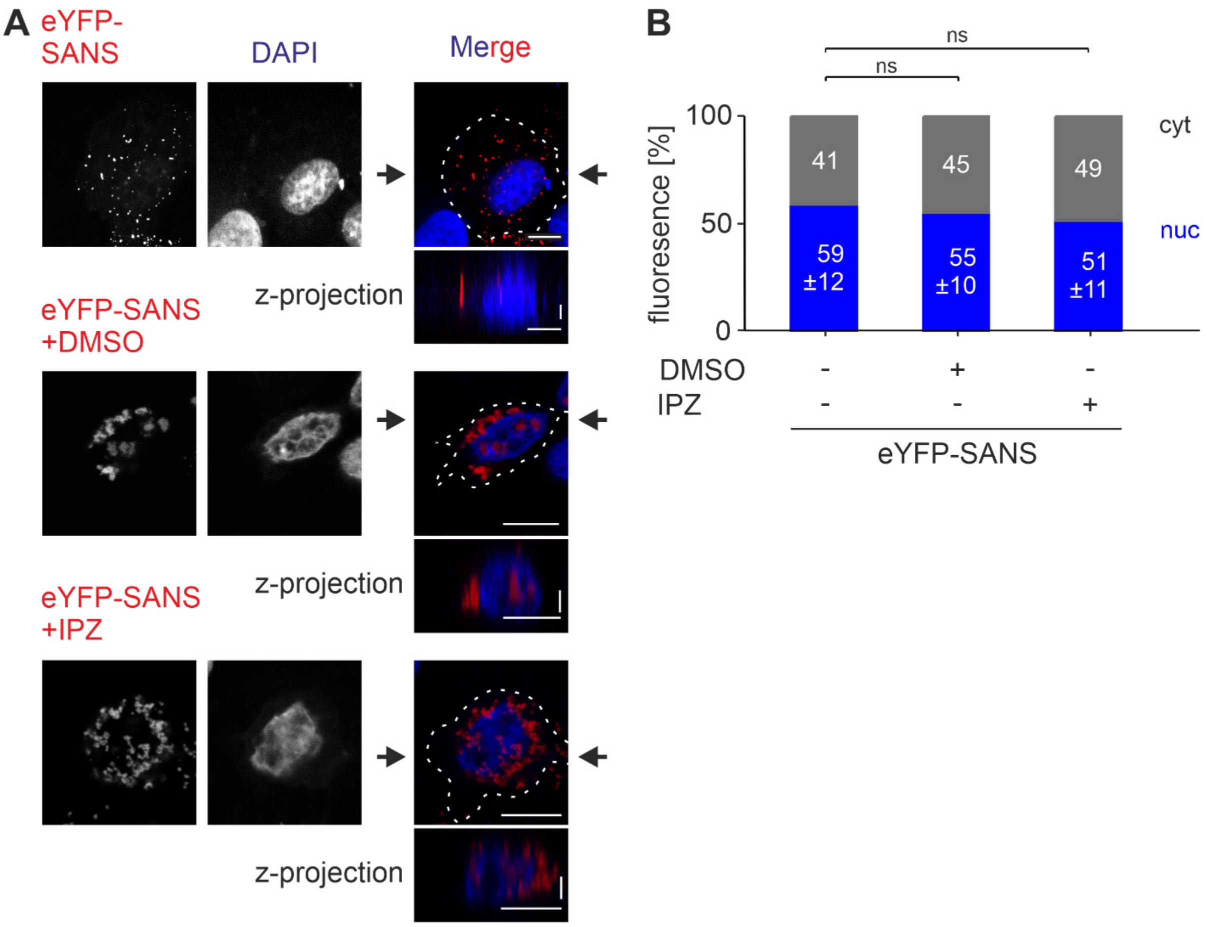

Figure S6. Nuclear localization of eYFP-SANS treated with Importazole. (A) Confocal microscopy of HeLa cells transfected with eYFP-SANS (red) and treated with DMSO or 40 µM Importin-β inhibitor Importazole (IPZ). **(B)** Quantification of (A) by CellProfiler. eYFP-SANS did not differ in its localization after IPZ treatment. Black arrows: position of Z-projections. Scale bars: horizontal = 10 µm; vertical = 2 µm. Data show mean values ± standard deviation from three independent experiments. Students t-test was performed for three independent experiments with a minimum of 75 cells; ; ns = not significant, p > 0,05.

Figure S7:

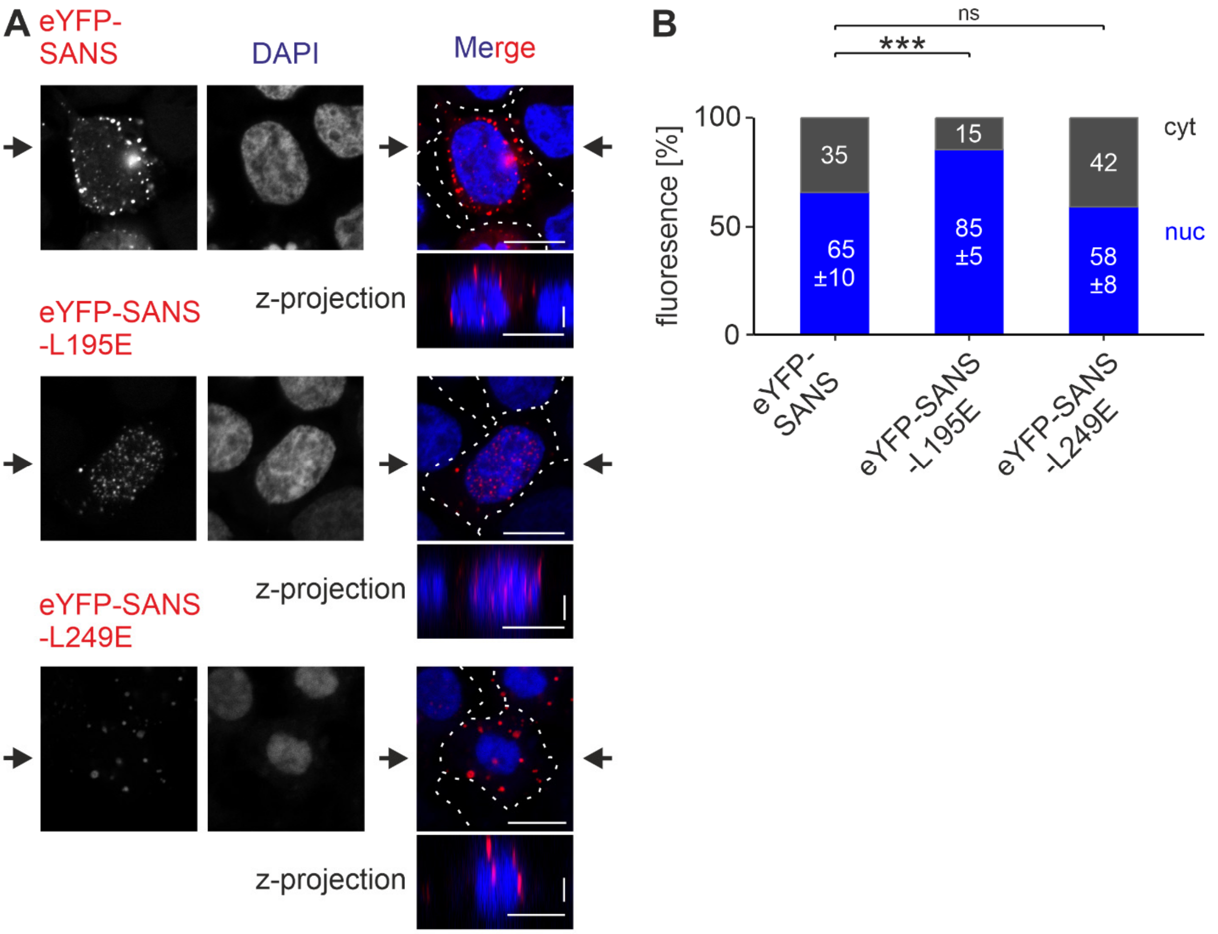

Figure S7. Localization of SANS NES mutants in HEK293T cells. (A) Confocal microscopy of HEK293T cells transfected with eYFP-SANS (red) or NES mutants, counterstained with DAPI. **(B)** Quantification of (A) by CellProfiler. eYFP-SANS^{L195E} was significantly enriched in the nucleus compared to eYFP-SANS. Black arrows: position of Z-projections. Scale bars: horizontal = 10 µm; vertical = 2 µm. Data show mean values ± standard deviation from three independent experiments. Students t-test was performed for 3 independent experiments with a minimum of 75 cells; ns = not significant, $p > 0{,}05$; *** = $p \leq 0.0009$.

Figure S8:

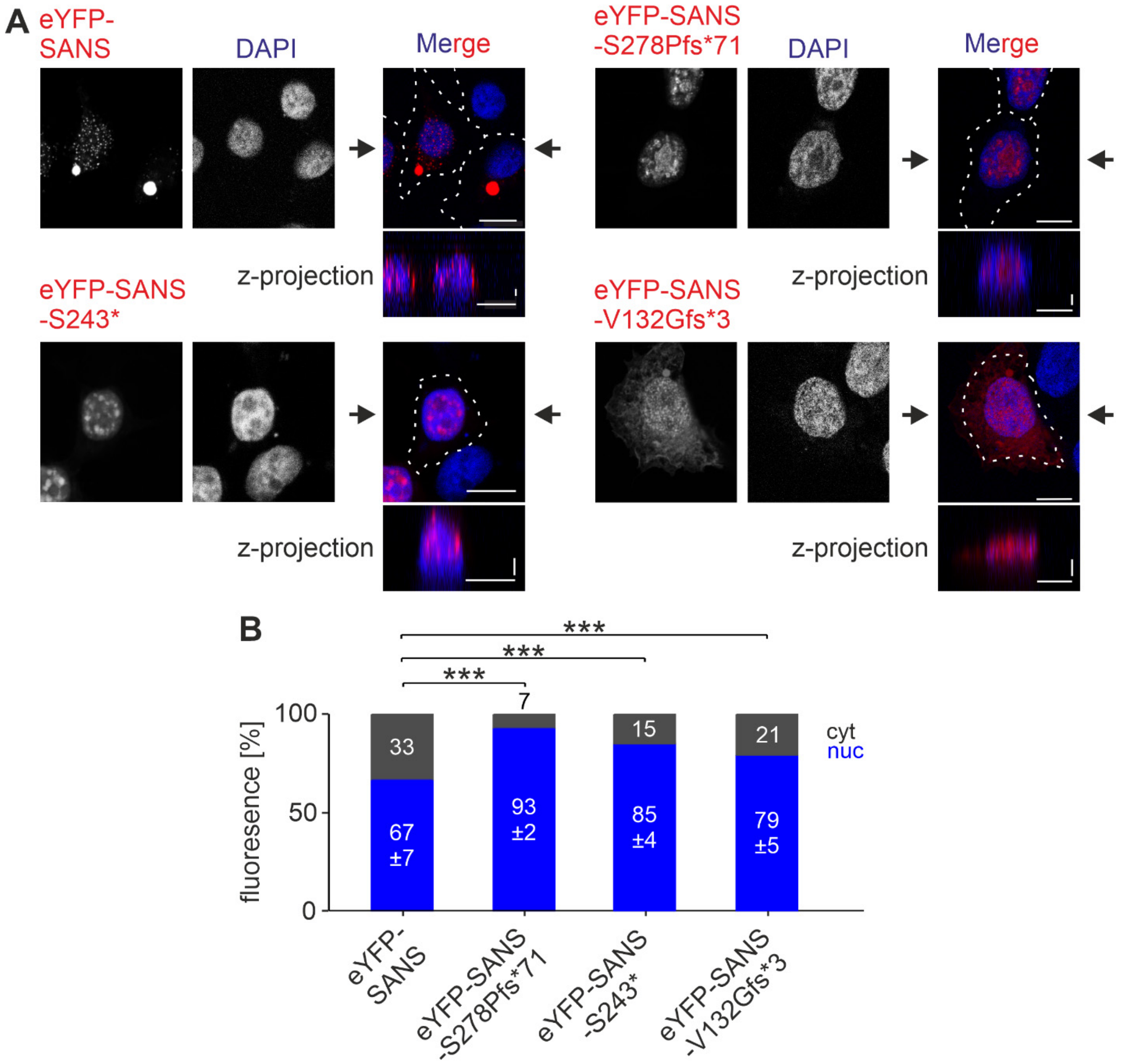

Figure S8. Localization of pathogenic variant of SANS in HEK293T cells. (A) Confocal microscopy of HEK293T cells transfected with eYFP-SANS (red), eYFP-SANS^{S278Pfs*71}, eYFP-SANS^{S243*} and eYFP-SANS^{V132Gfs*3}, counterstained with DAPI. **(B)** Quantification of (A) with CellProfiler. All pathogenic variants were significantly enriched in the nucleus. Black arrows: position of Z-projections. Scale bars: horizontal = 10 µm; vertical = 2 µm. Data show mean values ± standard deviation from three independent experiments. Students t-test was performed for 3 independent experiments with a minimum of 75 cells; *** = p ≤ 0.0009.

Figure S9:

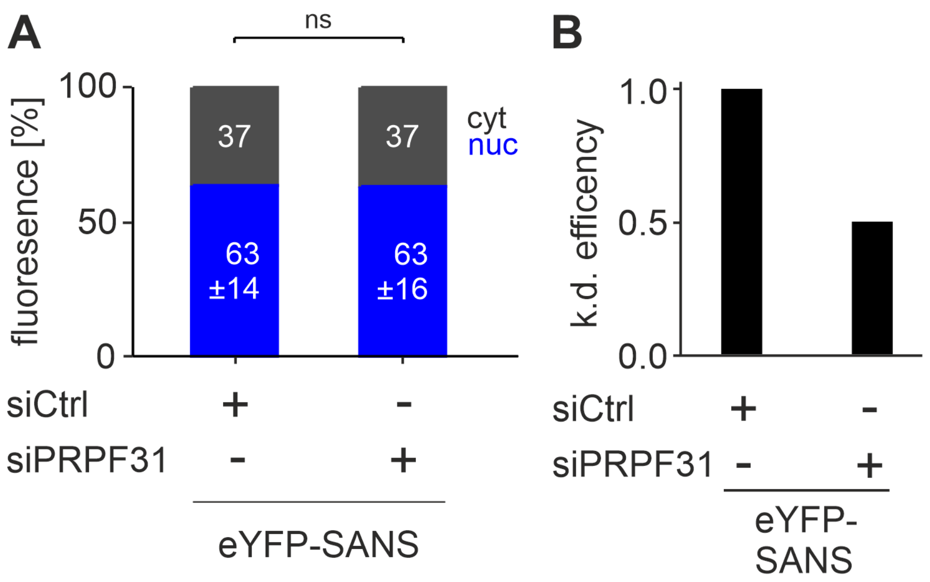

Figure S9. Localization of eYFP-SANS under siRNA-based PRPF31 knock down in eYFP-SANS transfected HeLa cells. (A) Quantification with CellProfiler demonstrated that siRNA-based knockdown of endogenous PRPF31 did not alter the subcellular localization of eYFP-SANS. Data show mean values ± standard deviation from three independent experiments. Students t-test was performed for 3 independent experiments with a minimum of 75 cells; ns = not significant, $p > 0,05$. **(B)** Knockdown efficiency of siCtrl and siPRPF31 with simultaneous transfection of eYFP-SANS.

1.2 Supplementary Tables

Table_S1.xlsx: GO-term analysis of SANS nuclear interactome from (Yildirim et al. 2021)

Table_S2.xlsx: Missense3D prediction of SANS^{K213E} and SANS^{L195E}

Table S3: Predicted nuclear export sequences (NES) of ANKS4B

NLS/NES	Sequence	Score	CRM1-class
NES_1$^{336-350}$	336-VEWEEDVVDATPLEV-350	0.191	1c
NES_2$^{338-352}$	338-WEEDVVDATPLEVFL-352	0.199	1c
NES_3$^{339-353}$	339-EEDVVDATPLEVFLL-353	0.205	1b
NES_4$^{346-360}$	346-TPLEVFLLSQHLEEF-360	0.181	2
NES_5$^{349-363}$	349-EVFLLSQHLEEFLPI-363	0.104	3
NES_6$^{362-376}$	362-PIFKREQIDLEALLL-376	0.251	2
